# Predicting hybrid fitness: the effects of ploidy and complex ancestry

**DOI:** 10.1093/genetics/iyaf242

**Published:** 2025-11-08

**Authors:** Hilde Schneemann, John J Welch

**Affiliations:** Department of Genetics, University of Cambridge, Cambridge CB2 3EH, United Kingdom; Department of Genetics, University of Cambridge, Cambridge CB2 3EH, United Kingdom

**Keywords:** hybridization, ploidy, heterosis, genetic interactions

## Abstract

Hybridization between divergent populations places alleles in novel genomic contexts. This can inject adaptive variation—which is useful for breeders and conservationists—or reduce fitness, leading to reproductive isolation. Most theoretical work on hybrids involves haploid or diploid hybrids between two parental lineages, but real-world hybridization is often more complex. We introduce a simple fitness landscape model to predict hybrid fitness with arbitrary ploidy and an arbitrary number of hybridizing lineages. We test our model on published data from maize (*Zea mays*) and rye (*Secale cereale*), including hybrids between multiple inbred lines, both as diploids and synthetic tetraploids. Quantitative predictions for the effects of inbreeding, and the strength of progressive heterosis, are well supported. Results suggest that the model captures the important properties of dosage and genetic interactions, and may help to unify theories of heterosis and reproductive isolation.

## Introduction

Hybridization occurs when individuals from genetically differentiated lineages mate and produce offspring. These hybrids carry novel combinations of alleles, exposing allelic effects in different genomic backgrounds (Burch et al. 2024; Peñalba et al. 2024). When alleles function well in their new background, hybridization can facilitate adaptation (e.g. Song et al. 2011; Pardo-Diaz et al. 2012; Abbott et al. 2013; Hedrick 2013; Kulmuni et al. 2023)—a fact exploited by breeders (East 1909; Shull 1909; Gowen 1952; Suneson 1956; Gerdes et al. 1999; Gur and Zamir 2004; Mackay et al. 2020; ter Steeg et al. 2022), and by conservationists (Genovart 2008; Chan et al. 2019). Conversely, when alleles function poorly, low fitness hybrids may form a barrier to genetic exchange, contributing to reproductive isolation and speciation (Dobzhansky 1937; Butlin 1987; Levin 1985; Hoskin et al. 2005). Hence, studying hybrids and predicting their fitness is important in several areas of biology.

Decades of empirical work has revealed recurrent patterns in hybrid fitness data, suggestive of common and general features of genetic interactions (Kölreuters 1766; Darwin 1859; Haldane 1922; Bateson 1978; Butlin 1987; Waser 1993; Trouve et al. 1998; Turelli and Moyle 2007; Schilthuizen et al. 2011; Wei and Zhang 2018; Dagilis et al. 2019; Coyne and Orr 2004, Chapter 8). Inspired by these data, theoretical work has investigated simple fitness landscape models, to see whether they can generate the patterns observed (Orr 1995; Barton 2001; Gavrilets 2004; Fraïsse et al. 2016; Simon et al. 2018; Satokangas et al. 2020; Schneemann et al. 2024). However, most theory has two major limitations. First, predictions apply only to haploid or diploid hybrids, while empiricists often study higher ploidies (Otto and Whitton 2000; Birchler 2013; Washburn and Birchler 2014; Clo and Kolář 2022). Second, most predictions apply to hybrids between just two parental lineages, while hybrids can have more complex ancestry—whether in nature (Harvey et al. 2019; Natola et al. 2022; Dean et al. 2024), or in agriculture (Busbice and Wilsie 1966; Groose et al. 1989; Bingham 1980; Riddle and Birchler 2008; Washburn et al. 2019). For example, in the Kerguelen islands, hybrid mussels (*Mytilus edulis* complex) contain ancestry from multiple populations of at least three species (Fraïsse et al. 2021); while on British organic farms, hexaploid winter wheat (*Triticum aestivum*), is grown as Composite Cross Populations, combining 20 inbred lines (Suneson 1956; Knapp et al. 2020).

Here, we consider a class of fitness landscapes used to study two-parent haploid and diploid hybrids by Barton (2001), Chevin et al. (2014), and De Sanctis et al. (2023). We generalize this model to hybrids of arbitrary ploidy, and with ancestry from an arbitrary number of parental lines. We then compare predictions to two extraordinary datasets, from maize (*Zea mays*; Yao et al. 2020), and rye (*Secale cereale*; Lundqvist 1966). Both data sets compare diploids and synthetic tetraploids of the same genotypes, and both contain hybrids between multiple inbred lines.

## Model

The aim of this work is to predict the fitness of a hybrid from a few measures of its genomic composition (Lande 1981; Hill 1982). For diploid hybrids between two parental lineages, A and B, the genomic composition can be quantified by the hybrid indices $h_{A}$ and $h_{B}=1-h_{A}$, defined as the proportion of alleles in the hybrid that derive from each parental lineage; and the ancestry heterozygosity $p_{\mathrm{AB}}$, defined as the proportion of loci with one allele from each lineage. To predict fitness from just these quantities, we need to assume that hybrids contain an effectively random sample of alleles from the parental populations (see also Appendix A: Derivation of main result). Even with this assumption, the predicted fitness might be an arbitrarily complex expression; this is because predictions need not be linear in $h_{A}$ and $p_{\mathrm{AB}}$, but might also include higher-order terms in e.g. $h_{A}^{3}$, $h_{A}^{2}p_{\mathrm{AB}}^{2}$ etc. Fitness landscape models allow us to make testable predictions about the relative sizes of these terms. In particular, previous work has derived the following prediction, which contains just one second-order term, and two free parameters:


(1) E(W)=hAWA+hBWB⏟fitnessofhybridizinglinesweightedbyancestry+14pABMAB⏟maskingduetoheterozygosity+hAhBIAB⏟epistaticinteractionsduetoadmixture

(Chevin et al. 2014; De Sanctis et al. 2023; Schneemann et al. 2024). In this expression, *W* is the fitness of the hybrid, which must be measured on a scale that is bounded above but unbounded below (e.g. the log number of expected offspring; Lynch and Walsh 1998, Chapter 10; Fraïsse et al. 2016; Schneemann et al. 2024), and $W_{A}$ and $W_{B}$ are the similarly transformed fitness of the original parental lineages in the environment where the hybrids were scored. The model also makes predictions about the parameters $M_{\mathrm{AB}}$ and $I_{\mathrm{AB}}$. In particular, it predicts that $M_{\mathrm{AB}}>0$, such that heterozygosity will always increase hybrid fitness. By contrast, $I_{\mathrm{AB}}$ can vary in sign (depending on the divergence and selection history of the parental lineages; see Chevin et al. 2014; Schneemann et al. 2020; De Sanctis et al. 2023) but is predicted to be bounded below at $I_{\mathrm{AB}}\geq -M_{\mathrm{AB}}$.

Biologically, equation (1) partitions the fitness effects of hybridization into (i) effects of the parental fitnesses, which are weighted by their contributions to hybrid ancestry; (ii) heterotic increases in fitness due to the masking effects of heterozygosity (Manna et al. 2011; Billiard et al. 2021; Schneemann et al. 2022); and (iii) fitness effects of admixture, which can be positive or negative, depending on the sign of $I_{\mathrm{AB}}$ (Slatkin and Lande 1994; Simon et al. 2018; Schneemann et al. 2020). The simplicity and inflexibility of equation (1) stem from its origin in a simple model of quadratic optimizing selection on additive quantitative traits (Lande 1976; Barton 2001). This model implies that only interactions between pairs of alleles contribute to fitness variation (Hill 1982; Martin et al. 2007). Nonetheless, it generates a rugged genotypic fitness landscape, with variable fitness dominance and epistasis (Martin et al. 2007; Manna et al. 2011; Hwang et al. 2017), and it might make successful predictions even when the phenotypic model is not taken literally (Martin 2014). A full derivation is found in Appendix A: Derivation of main result) and extensions that allow for, e.g. directional- or under-dominance are found in Schneemann et al. (2022) and De Sanctis et al. (2023).

### Extension to complex ancestry and arbitrary ploidy

Equation (1) applies to hybrids between two parental lineages with diploid segregation. However, it is easily generalized with a change in notation. To see this, let us redefine $h_{A}$ as the proportion of alleles that descend from lineage A at a single locus; so that the hybrid index is its expected value across loci, now denoted $\left\langle h_{A}\right\rangle$. For a heterozygous locus, $h_{A}$ and $h_{B}$ are both 1/2, such that the expected heterozygosity across loci is $p_{\mathrm{AB}}=4\langle h_{A}h_{B}\rangle$. Finally, if we number the parental lineages from i=1⋯P, then with P=2 equation (1) can be written as


(2) $$E(W)=\sum_{i}^{P}\langle h_{i}\rangle W_{{\mathrm{Par}}_{i}}+\sum_{i,j>i}^{P}\langle h_{i}h_{j}\rangle M_{ij}+\sum_{i,j>i}^{P}\langle h_{i}\rangle \langle h_{j}\rangle I_{ij}.$$

In Appendix A: Derivation of main result, we show that equation (2) applies equally to hybrids containing ancestry from an arbitrary number of parental lineages, *P*, and with arbitrary ploidy, *K*.

Although *K* does not appear explicitly in equation (2), the equation does allow us to distinguish between two possible consequences of polyploidization. First, a change in ploidy might alter the fitnesses of the parental genotypes, $W_{{\mathrm{Par}}_{i}}$, and the parameters $M_{ij}$ and $I_{ij}$. In the current framework, the resulting changes could only be predicted if some property of the underlying phenotypic effects remained unchanged after polyploidization. For example, if the homozygous effects of alleles remained unchanged, then $W_{{\mathrm{Par}}_{i}}$, $M_{ij}$, and $I_{ij}$ would also remain unchanged. If, by contrast, the individual allelic effects were unchanged, then $W_{{\mathrm{Par}}_{i}}$, $M_{ij}$, and $I_{ij}$ would all scale with $K^{2}$ (see Appendix A: Derivation of main result equations (A14)–(A16)). A second set of predictions involve the measures of ancestry, $\left\langle h_{i}\right\rangle$, $\left\langle h_{i}h_{j},\right\rangle$ and $\left\langle h_{i}\rangle \langle h_{j}\right\rangle$. How these quantities change across generations will depend on the mode of segregation (e.g. disomic, tetrasomic), and thereby on the ploidy (Otto and Whitton 2000; Riddle et al. 2006; Soltis et al. 2014). We can test predictions about these changes, whatever the other effects of polyploidization.

## Testing the predictions

In the remainder of the article, we test the predictions of equation (2) against the data of Yao et al. (2020) and Lundqvist (1966). While each study reported plausible correlates of vigor and reproductive success, neither measured fitness directly. This is an issue because proxies for fitness have no natural scale of measurement, and different transformations of the data will give different fits to the model (Fraïsse et al. 2016; Carlson et al. 2025). While such flexibility is sometimes useful, allowing us to choose the best-fitting transformation (Lynch and Walsh 1998, Chapter 11), here we use a more stringent approach. First, before undertaking any analyses, we chose the reported trait that seemed to us the most suitable proxy for fitness. We then transformed the trait measurements to better meet the assumptions of the statistical tests, but prior to the model fitting itself, and without assuming the adequacy of equation (2) (see below for details). Before each reanalysis, we derive the additional results required to fit the model to each data set.

### Inbreeding depression with selfing

While initial F1 hybrids often see heterotic gains in fitness and vigor, subsequent inbreeding tends to reduce the gains, implying a role for heterozygosity. A major goal of Yao et al. (2020) was to explore the effects of ploidy on this loss of heterosis under inbreeding (Muller 1914; Haldane 1930), comparing diploids and tetraploids. One complication is that tetraploids—even with P=2 parents—contain two types of heterozygote: balanced (AABB) and unbalanced (AAAB or ABBB), which might contribute differently to fitness (Lundqvist 1966; Ronfort 1999; Birchler and Veitia 2012). Equation (2) assumes that all such effects can be captured by interactions between pairs of alleles, so that we only need to track the genome-wide average of $h_{A}h_{B}$. For balanced and unbalanced heterozygotes, we have $h_{A}h_{B}=\frac{2}{4}\frac{2}{4}$ and $h_{A}h_{B}=\frac{1}{4}\frac{3}{4}$, respectively, and so


(3) $$\langle h_{A}h_{B}\rangle =\frac{4}{16}\mathrm{Pr}(\mathrm{AABB})+\frac{3}{16}\mathrm{Pr}(\mathrm{AAAB}\cup \mathrm{ABBB}),\;K=4,\;P=2$$

which is a weighted sum of both types of heterozygote. To see how $\left\langle h_{A}h_{B}\right\rangle$ changes under inbreeding, let us note its relation to Wright’s inbreeding coefficient, *F* (Wright 1922): the probability that two alleles chosen without replacement from the same locus, are identical by descent (i.e. descend from the same parental lineage). From this definition, it follows that


(4) $$\langle h_{A}h_{B}\rangle =\frac{1}{2}\left(1-\frac{1}{K}\right)(1-F).$$

After *t* generations of selfing, the inbreeding coefficient changes at a constant rate *β*, via


(5) $$F_{t}=(1-\beta )+\beta F_{t-1}$$

(Wright 1969; Crow and Kimura 1970). To calculate *β*, we then assume even ploidy and random chromosome segregation during meiosis without double reduction (Gallais 2003), which yields


(6) $$\beta =1-\frac{1}{2K-2}$$

(Hardy 2015). Combining these results, we have


(7) $$\begin{array}{rl} \langle h_{A}h_{B}\rangle_{t} & =\beta \langle h_{A}h_{B}\rangle_{t-1}\\ & =\beta^{t}\langle h_{A}h_{B}\rangle_{0} \end{array}$$

Since selfing will not change the expected mix of ancestries, $\left\langle h_{A}\right\rangle$ and $\left\langle h_{B}\right\rangle$, we then obtain the final result for the total decrease in fitness


(8) $$\begin{array}{rl} E\left(W_{t}-W_{0}\right) & =\left(\langle h_{A}h_{B}\rangle_{t}-\langle h_{A}h_{B}\rangle_{0}\right)M_{\mathrm{AB}}\\ & =\left(\beta^{t}-1\right)\frac{1}{4}M_{\mathrm{AB}}, \end{array}$$

where *β* is given by equation (6), and we have used the fact that $\langle h_{A}h_{B}\rangle_{0}=1/4$ in the initial F1 hybrid.

### Testing the predictions in maize

To test the prediction of equation (8), we used data from Yao et al. (2020). These data comprise hybrids of four inbred lines of maize (*Zea mays* lines A188, Oh43, W22, and B73), here labeled A, B, C, and D. In the main text, we consider only the A×B and C×D crosses, which were generated in both diploid and tetraploid form, via nitrous oxide gas treatment of the diploid zygotes (Kato and Birchler 2006; Yao et al. 2020). Appendix B: Details of reanalysis of Yao et al. (2020) maize data discusses attempts to interpret the AB×CD double cross and crosses only available as diploids.

**Fig. 1.**
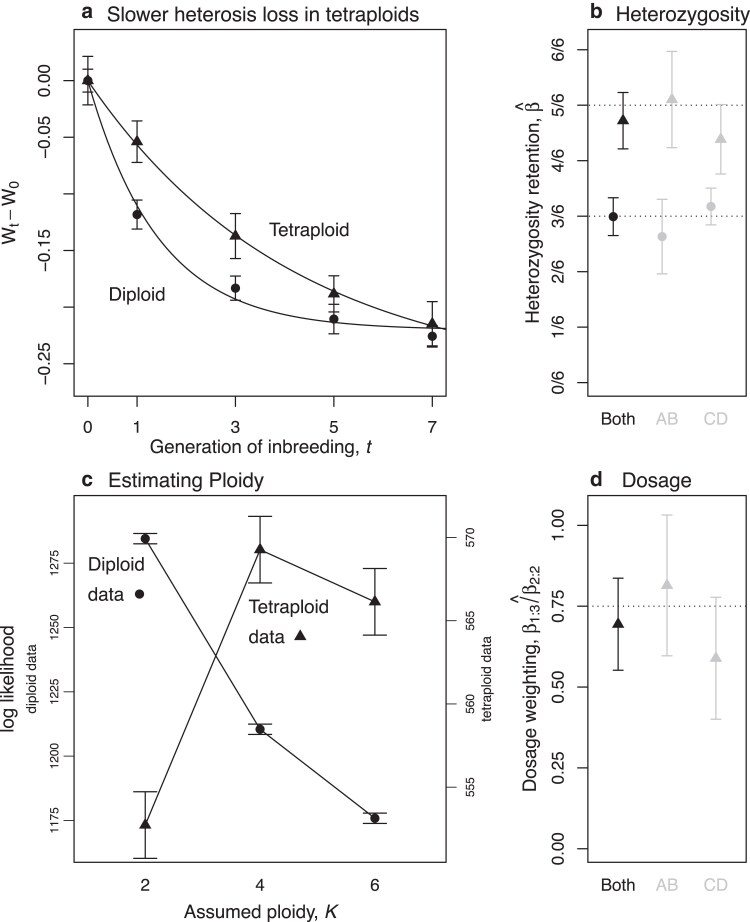
Reanalysis of hybrid maize data from Yao et al. (2020). a) The decline in fitness over 7 generations of inbreeding is slower in tetraploid (▴) than in matched diploid (∙) maize crosses. Points and bars show means and standard errors for ear length, measured as deviations from the appropriate F1, then pooled over crosses, and transformed to minimize the skew around the within-generation means (see Appendix B: Details of reanalysis of Yao et al. (2020) maize data). Lines show the least-squares fit of the nonlinear model of equation (B1) (Baty et al. 2015) with *β* fixed at its expected value. b) Estimates of the parameter *β*, which captures the retention of heterozygosity, with 95% confidence intervals (Baty et al. 2015). Estimates of *β* for both pooled and individual crosses match predictions of β=1/2 for diploids, and β=5/6 for tetraploids (equation (6)). c) Estimates of ploidy from model comparison with K=2,4, or 6 indicate that the correct ploidy gives the best likelihood for both the pooled diploid and tetraploid data (left and right *y*-axis, respectively). Error bars indicate 2 units of log likelihood. d) Fitting equation (9), estimates the different effects of balanced and unbalanced heterozygotes in tetraploids, and agrees with predictions of $\beta_{13}/\beta_{22}=3/4$ (confidence intervals on the ratio used the “delta method”; Fox et al. 2001; Fox and Weisberg 2019).

Yao et al. selfed these initial F1 for seven generations (with deviations from the cross- and ploidy-specific F1 reported at generations 0, 1, 3, 5, and 7 in five replicate experiments). We chose ear length as the available trait closest to overall plant fitness or productivity. We then Box–Cox transformed the reported measurements to minimize the skewness of the data around their means for each generation (see Appendix B: Details of reanalysis of Yao et al. (2020) maize data for full details), but without assuming the adequacy of equation (8). We then fit equation (8) using standard least-squares nonlinear regression (Baty et al. 2015). The full model includes one *β* value per ploidy, and an $M_{ij}$ value for each ploidy/cross combination. However, without raw F1 measurements, only the *β* values are readily interpretable. Figure 1 shows these *β* values estimated either from individual crosses, or from data pooled from both crosses.

Figure 1a shows results of fitting data from the two ploidies. As expected, the tetraploids lose fitness at a slower rate, matching the predictions of equation (6) that β=1/2 for diploids and β=5/6 for tetraploids (Fig. 1b). Conversely, we can directly estimate ploidy from data of this kind by comparing the model fit under different values of *K* (Fig. 1c). Indeed, we find that the true values K=2 and K=4 provide the best fit for the diploid and tetraploid data, respectively. With the tetraploid data, we can go further, and estimate the weights in equation (3), which determine the effects of dosage, via the relative contributions of balanced and unbalanced heterozygotes. In this case, with K=4 and P=2, we fit the model


(9) $$\begin{array}{rl} E\left(W_{t}-W_{0}\right) & =[\beta_{2\;:\;2}\left({\mathrm{Pr}}_{t}(\mathrm{AABB})-1\right)\\ & \;+\beta_{1\;:\;3}{\mathrm{Pr}}_{t}(\mathrm{AAAB}\cup \mathrm{ABBB})]M_{\mathrm{AB}}, \end{array}$$

where


(10) $$\begin{array}{rl} {\mathrm{Pr}}_{t}(\mathrm{AABB}) & =\frac{1}{2}\left({\left(\frac{5}{6}\right)}^{t}+{\left(\frac{1}{6}\right)}^{t}\right)\\ {\mathrm{Pr}}_{t}(\mathrm{AAAB}\cup \mathrm{ABBB}) & =\frac{2}{3}\left({\left(\frac{5}{6}\right)}^{t}-{\left(\frac{1}{6}\right)}^{t}\right) \end{array}$$

(which follow from recursions in Haldane 1930). Then, we can estimate their ratio (but not their individual values), which equation (3) predicts to be $\beta_{1\;:\;3}/\beta_{2\;:\;2}=(\frac{1}{4}\frac{3}{4})/(\frac{2}{4}\frac{2}{4})=3/4$. The results, shown in Fig. 1d, demonstrate that the estimates of this ratio from the data are consistent with the prediction of our model.

### Progressive heterosis with multiple parents

**Fig. 2.**
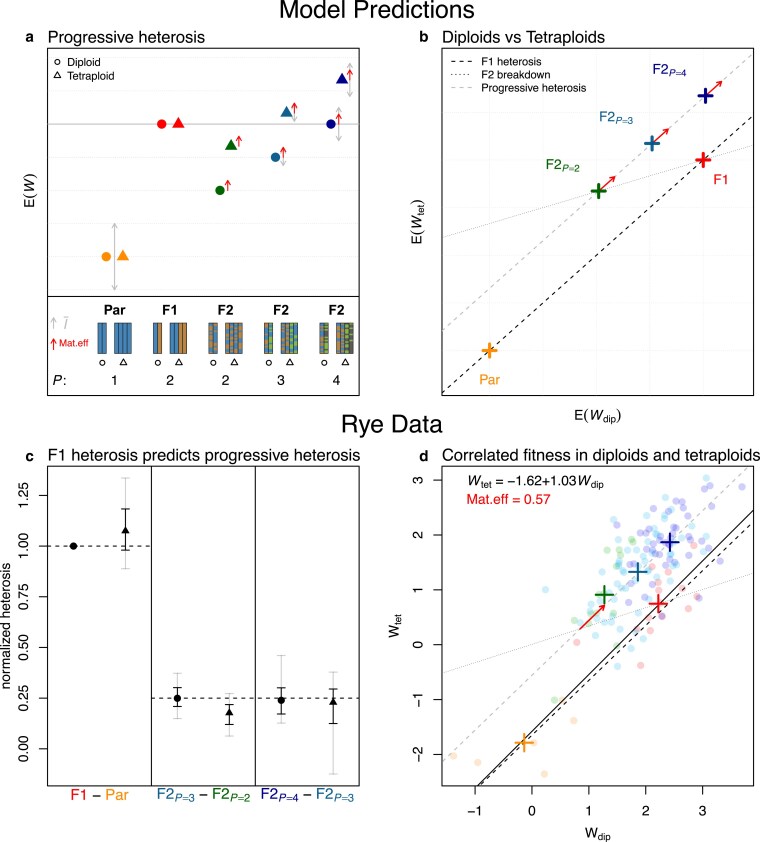
Reanalysis of hybrid rye data from Lundqvist (1966). a) Predicted fitness for different crosses in diploids (∙) and tetraploids (▴), with ancestry from P=1,2,3, or 4 parental lines (equation (2)). The diagram assumes equal fitness for the parents and F1 for visual clarity, and with this assumption, ploidies differ only in their F2 breakdown (fitness reduction between F1 and F2; equation (12)). Fitness epistasis, $\bar{I}$ (faint double-sided arrows), can vary in sign, and affect both F1 heterosis (improvement between Parents and F1; equation (11)) and progressive heterosis (improvement from adding parents to the F2; equation (16)). A maternal affect (upward facing red arrows) may increase the fitness of all F2, because they were grown from fitter F1 mothers. b) Slopes that indicate F1 heterosis and progressive heterosis should match (equation (16); dashed lines); while the F2 breakdown slope should be 1/3 smaller (equation (12); dotted line), but maternal effects make the latter difficult to test. c) F1 heterosis in diploids accurately predicts the progressive heterosis, in both diploids and tetraploids (equation (16)). Results show the difference in $\bar{W}$ for the crosses indicated, normalized by the difference between diploid parents and F1. Error bars are jackknife resamples, removing any cross with ancestry from each of 6 inbred lines (black bars), or each of 15 pairs of lines (faint bars). d) Rye data, with total kernel yield transformed to maximize the correlation between ploidies for the Parent and F1 measurements (see Appendix C: Details of reanalysis of Lundqvist (1966) rye data), and individual crosses (∙) compared to means of cross types (+). The best-fit SMA regression (Warton et al. 2012) has a slope close to 1 (solid line), implying that polyploidization little changed the $M_{ij}$ and $I_{ij}$ parameters.

A major goal of Lundqvist (1966) was to explore the effects on heterosis both of ploidy and multiparent ancestry. Equation (2) assumes that these will also be predictable from ancestry measures involving pairs of alleles. Figure 2a shows the predictions from equation (2) for diploids and tetraploids up to the F2 generation. To clearly see the patterns of interest in this diagram, we assume constant fitness across ploidies for both the parents and F1, although neither is assumed in the analysis below.

Three main patterns are evident in Fig. 2a. First, there is F1 heterosis under both ploidies: a large increase in fitness between the parents and F1. As shown by Hill (1982) this heterosis can involve both dominance and epistasis, and if we average over all F1, we have


(11) $$E\left({\bar{W}}_{F1}-{\bar{W}}_{\mathrm{Par}}\right)=\frac{1}{4}\left(\bar{M}+\bar{I}\right),$$

where $\bar{M}$ and $\bar{I}$ are averages over all pairs of parental lineages. It follows that heterosis will differ among ploidies only if $\bar{M}$ and $\bar{I}$ also differ, and given I≥−M that heterosis cannot be negative i.e. that F1 are at least as fit as their parents (Barton 2001; Fraïsse et al. 2016; Schneemann et al. 2022).

The second pattern in Fig. 2a is the loss of fitness between the F1 and the F2 with ancestry from two parental lines (P=2). As we saw in the previous section, the strength of this F2 breakdown depends on ploidy because of the ploidy-dependent rate of heterozygosity loss. Using equations (6) and (8), we have


(12) $$\begin{array}{rl} \frac{E{\left({\bar{W}}_{F2}-{\bar{W}}_{F1}\right)}_{\mathrm{tet}}}{E{\left({\bar{W}}_{F2}-{\bar{W}}_{F1}\right)}_{\mathrm{dip}}} & =\frac{(5/6-1){\bar{M}}_{\mathrm{tet}}}{(1/2-1){\bar{M}}_{\mathrm{dip}}}=\frac{1}{3}\frac{{\bar{M}}_{\mathrm{tet}}}{{\bar{M}}_{\mathrm{dip}}}. \end{array}$$

The final pattern in Fig. 2a is the steady increase in F2 fitness with the number of parental lineages, *P*. Sometimes, F2 fitness exceeds the F1, a phenomenon known as progressive heterosis (Washburn and Birchler 2014). To understand this, let us first consider the average fitness of all F2 derived from *P* parental lineages:


(13) $$E\left({\bar{W}}_{{F2}_{P}}\right)={\bar{W}}_{\mathrm{Par}}+\left(\sum_{i,j>i}^{P}\langle h_{i}h_{j}\rangle \right)\bar{M}+\left(\sum_{i,j>i}^{P}\langle h_{i}\rangle \langle h_{j}\rangle \right)\bar{I},$$

where the sums in brackets would be identical for any *P*-parent F2. The second sum is found to be $\sum_{i,j>i}^{P}\langle h_{i}\rangle \langle h_{j}\rangle =\frac{P}{16}+\frac{1}{8}$, while the first sum equals the right-hand-side of equation (4). In the F2, the inbreeding coefficient is


(14) $$\begin{array}{rl} F & =\left(1-p_{d}\right)\phi_{s}+p_{d}\phi_{d}=\left(1-\frac{K/2}{K-1}\right)\frac{K/2-1}{K-1}+\frac{K/2}{K-1}\left(1-\frac{P}{4}\right), \end{array}$$

where $p_{d}$ is the probability that two alleles, drawn at random from an F2 locus, derive from different F1 gametes; and $\phi_{s}$ ($\phi_{d}$) is the probability that alleles drawn from the same gamete (different gametes) are identical by descent. Substituting this result into equation (4) gives us $\sum_{i,j>i}^{P}\langle h_{i}h_{j}\rangle =\frac{P}{16}+\frac{1}{8}\frac{K-2}{K-1}$. We can now compare the fitness of the F1 and F2 generation (with P∈2,3,4):


(15) $$\begin{array}{rl} E\left({\bar{W}}_{{F2}_{P}}-{\bar{W}}_{F1}\right) & =\frac{1}{8}\left[\frac{K-2}{K-1}\bar{M}+\bar{I}\right]-\frac{1}{4}\left[1-\frac{P}{4}\right]\left(\bar{M}+\bar{I}\right). \end{array}$$

This shows that progressive heterosis is most likely to appear in four-parent F2 hybrids, where the second term disappears. Still, in diploids (K=2) progressive heterosis will appear only when epistasis is positive ($\bar{I}>0$); while in tetraploids (K=4) progressive heterosis will always appear, unless epistasis is very strong and negative, such that $\bar{I}<-\frac{2}{3}\bar{M}$. Finally, we can relate the strength of the progressive heterosis to the F1 heterosis, by noting that


(16) $$\begin{array}{rl} E\left({\bar{W}}_{{F2}_{P+1}}-{\bar{W}}_{{F2}_{P}}\right) & =\frac{1}{16}\left(\bar{M}+\bar{I}\right)\;P\in 2,3\\ & =\frac{1}{4}E\left({\bar{W}}_{F1}-{\bar{W}}_{\mathrm{Par}}\right). \end{array}$$

So for any ploidy, the fitness gain from adding an additional parent to the F2, is equal to a quarter of the original F1 heterosis. Note that the first line of equation (16)—the linear change with *P*—holds quite generally, because the total number of heterozygous loci changes linearly for both ploidies (Lundqvist 1966). However, the second line—which relates F1 heterosis and progressive heterosis—holds in polyploids only under our particular weighting of the different types of heterozygote, and thus presents a more specific test of our model. All three predictions (equations (11), (12), and (16)) are summarized in Fig. 2b.

### Testing the predictions in rye

To test these predictions, we use a classic dataset from Lundqvist (1966), involving six inbred parental lines of diploid rye (*Secale cereale*; steel variety, lines 19–23 and 25), here labeled A–F. Unlike the modern data of Yao et al. (2020) and Lundqvist (1966) studied only two generations of hybridization, and reported only means for each cross; but the dataset includes not only the 6 parental lines and the $\left(\begin{array}{c} 6\\ 2 \end{array}\right)=15$ possible F1, but also the $\left(\begin{array}{c} 15\\ 2 \end{array}\right)+15=120$ possible F2. These data allow us to test the effects of complex ancestry, because the F2 had ancestry from either P=2, 3 or 4 parents, depending on whether it was generated by selfing an F1 or crossing F1s with or without a shared parent (e.g. AB×AC or AB×CD). Moreover, we can also compare results for diploids and tetraploids, since colchicine treatment was used to generate synthetic tetraploids of the six inbred lines; and of the 141 crosses, only a single F1 was absent in tetraploid form. Out of the traits measured, we used total kernel yield as the best-available proxy for plant fitness. As we only have the means, we cannot unskew the data as before. However, we do have six parental lines and 14 F1s, such that we can now account for the fitness effect of polyploidization *per se*. Using only these fixed genotypes, we Box–Cox transformed the data to maximize the fit ($r^{2}$) of a Standardized Major Axis (SMA) regression between the diploid and tetraploid measurements (Warton et al. 2012). We also used this best-fit regression to impute a value for the missing tetraploid F1 (see Appendix C: Details of reanalysis of Lundqvist (1966) rye data for full details). The resulting data are plotted in Fig. 2d.

The first notable result in Fig. 2d is that the best-fit regression line between the diploid and tetraploid fixed genotypes (solid line), is very close to a 1:1 line, but with a nonzero intercept (dashed line). This result is consistent with polyploidization inducing a constant fitness cost for all genotypes, but with the $M_{ij}$ and $I_{ij}$ parameters remaining constant—a hypothesis supported by model selection using the complete data set of 282 crosses (see Appendix C: Details of reanalysis of Lundqvist (1966) rye data for full details).

**Fig. C3.**
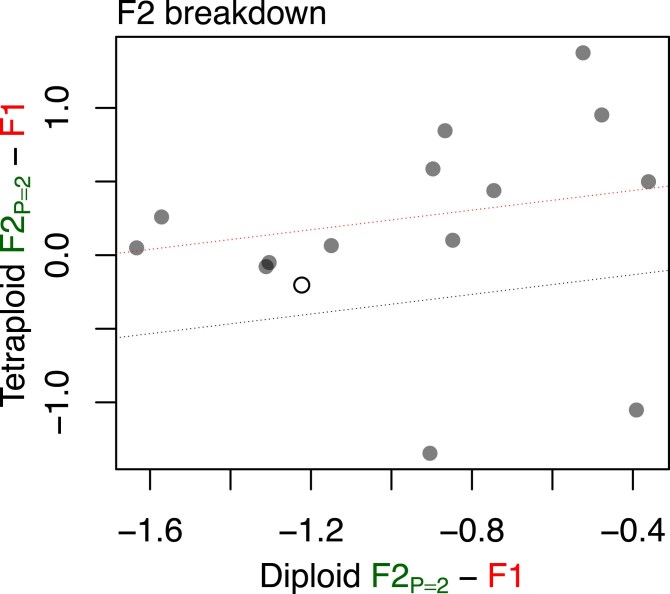
F2 breakdown in rye. All two-parent F2 have lower fitness than F1 in diploids, but not in tetraploids. The red (upper) and gray (lower) dotted lines show the expected relationship between the degree of F2 breakdown in diploids and tetraploids, following from equation (12), with and without the estimated maternal effect (slope=1/3, intercept=0.57 or 0). The open point shows the interpolated tetraploid value (see Fig. C1d).

The second observation from Fig. 2d is the failure of the prediction of hybrid breakdown (equation (12)). In fact, the two-parent tetraploid F2 are generally fitter than the equivalent F1 (see also Fig. C3). Lundqvist (1966) also remarked on this surprising aspect of his data, and speculated that a strong maternal effect might have provided a fitness boost to all F2 plants (who came from high-fitness F1 mothers), relative to the F1 and parental plants (which came from low-fitness inbred mothers). Red arrows in Fig. 2a,b show how a maternal effect would affect our predictions. Note that, even if the effect applied equally across ploidies, it might still remove hybrid breakdown completely only in the tetraploids (as observed). We could not test directly for a maternal effect, since crosses were not generally made in both directions (Lundqvist 1966). However, given our other assumptions, models with a constant maternal effect were preferred (see Appendix C: Details of reanalysis of Lundqvist (1966) rye data).

Finally, in Fig. 2c, we test the prediction of equation (16). Given the apparent constancy of the $M_{ij}$ and $I_{ij}$ parameters across ploidies, we used the observed heterosis in diploid F1, to predict the effects of adding parents to F2 of both ploidies. While tetraploid two-parent F2 were slightly fitter than predicted, the quantitative agreement was otherwise good. For example, the strength of F1 heterosis in diploids gave a remarkably accurate prediction for the benefit of adding a fourth parent in tetraploids.

## Discussion

We have presented a fitness landscape model to predict the fitness of hybrids of arbitrary ploidy and with an arbitrary mix of ancestry. Our results extend previous work on this model that applied only to two-parent haploids or diploids (Barton 2001; Chevin et al. 2014; De Sanctis et al. 2023) and allopolyploids with effectively diploid segregation (see the reanalysis of *Brassica* data from Hauser et al. 1998 by Schneemann et al. 2024). The model presented here applies under any mode of segregation as long as one can measure or predict the expected covariance in ancestry. Our model can also be seen as a generalization of classical single-locus inbreeding theory (Wright 1977, pp. 21–25) incorporating pairwise epistatic effects. As we discussed in previous work, these epistatic effects tend to become important incompatibilities in hybrids between divergent parents (Fraïsse et al. 2016; Simon et al. 2018; Schneemann et al. 2022; De Sanctis et al. 2023). As a result, this model can be used to study heterosis as well as reproductive isolation. The model can also be seen as a special case of autopolyploid quantitative genetics models that can be applied to any trait (e.g. Fisher 1965; Busbice and Wilsie 1966; Bennett 1976; Gallais 2003). Compared to these more general models, our focus on fitness (or proxies thereof) and assumptions of the underlying phenotypic model (i.e. quadratic fitness function and additivity of phenotypic effects) restrict the number of parameters and add an interpretation to their relative values in terms of the divergence history (De Sanctis et al. 2023). Our predictions about the masking effects of different types of heterozygote (e.g. AAAB vs. AABB) follow directly from these assumptions and gave a good fit to the data analyzed here (e.g. Figs. 1d and 2c).

### Explaining heterosis

We applied our model to two published data sets (Lundqvist 1966; Yao et al. 2020), both of which show heterosis, i.e. increased fitness for early generation hybrids (Kölreuters 1766; Darwin 1859; Gowen 1952; Birchler 2013; Muraro et al. 2022). There are longstanding debates about the causes of heterosis—and especially the adequacy of the simplest theory: that the parental lines fixed recessive deleterious alleles, whose effects are masked in the hybrids (Bruce 1910; Keeble and Pellew 1910; Crow 1948; Lippman and Zamir 2007; Birchler 2013; Washburn and Birchler 2014). The same process of masking—sometimes called “the dominance theory” or “the complementation theory”—could, in principle, explain the progressive heterosis observed in polyploids with multiparent ancestry (Fig. 2a,b; Jones 1918; Stringfield 1950; Bingham 1980; Groose et al. 1989). Our fitness landscape model offers a generalization of the classical masking theory, and may help to resolve some of these debates.

For example, if heterosis is caused by masking of deleterious alleles, then we could expect to select out these alleles, thereby “fixing” the heterosis—but this has not always proven possible (Jones 1917; Vetukhiv 1954; Birchler 2003; Koltunow and Tucker 2003; Washburn et al. 2019). Here, we have shown that F1 heterosis (equation (11)), and progressive heterosis (equation (15)) can both arise via masking, even when there are epistatic fitness interactions between the parental alleles. Nevertheless, with epistasis, “deleterious” alleles might be deleterious only in some genetic backgrounds (Hwang et al. 2017; Xie et al. 2022), and this could make them difficult to purge. Thus, our model may help to reconcile the observation of heterosis arising from masking, with the difficulty of fixing heterosis through selection on hybrid populations (Birchler 2013).

More directly, the magnificent data of Yao et al. (2020) were originally reported as evidence against the masking theory, because no evidence was found that diploids and tetraploids lost fitness at different rates. Here, by contrast, we did find a difference in the predicted direction (Fig. 1b). One explanation is that the form of the relationship is nonlinear (equation (B1)), and so unlikely to be detected by fitting a standard linear model (Yao et al. 2020), especially if data are not transformed to meet the assumptions of the model fitting (see Appendix B: Details of reanalysis of Yao et al. (2020) maize data).

Nonlinearity and scales of measurement are relevant for a second debate about the masking theory. If deleterious mutations are successfully removed, then the theory predicts a reduction in the potential for heterosis, which is not always observed (Duvick 2005; Troyer and Wellin 2009). However, the quantitative change in the heterosis with selective improvement of the parents will depend on the fitness proxy and data transform used. So, for example, yield might show increasing heterosis, while log yield shows decreasing heterosis. As such, observed changes in heterosis (Duvick 2005; Troyer and Wellin 2009) are difficult to use as evidence either for or against the masking theory (Birchler 2013; Washburn et al. 2019; see Appendix D: Heterosis and parental improvement for more details). Whatever the outcome of these debates, the fitness landscape model used here, incorporating, as it does, different types of gene action (Manna et al. 2011; Sellis et al. 2011) may facilitate quantitative analysis of hybrid fitness data and aid progress towards a unifying theory of heterosis (Birchler 2013).

### Polyploidization and hybridization

While both reanalyses presented here (Figs. 1 and 2) concern heterosis, we hope the theoretical results will apply more widely. If hybridization can contribute to biodiversity by injecting adaptive variation (Kulmuni et al. 2023; Peñalba et al. 2024), it can also do so by failing—thereby increasing reproductive isolation. Polyploidization may play a special role in both the positive and negative processes. For example, polyploid hybrids could have enhanced adaptive potential—not only by better masking their ancestors’ deleterious mutations (as discussed above) but also by combining their adaptations in a single individual, or by releasing pleiotropic constraints via specialization of the subgenomes (Soltis and Soltis 2000; te Beest et al. 2011; Van de Peer et al. 2017). On the other hand, ploidy differences can pose instant and strong barriers to gene exchange (Marks 1966; Otto and Whitton 2000; Turelli et al. 2001; Hegarty and Hiscock 2004; Soltis et al. 2014) and can themselves be caused by hybridization (Masterson 1994; Lewis 2012). Indeed, these are all possible explanations for the abundance of polyploids in nature.

In our approach, the positive and negative effects of hybridization are governed by the size and magnitude of the parameters (i.e. the $M_{ij}$ and $I_{ij}$), and the underlying phenotypic approach allows us to predict their changes under different modes of evolutionary divergence (Chevin et al. 2014; Simon et al. 2018; Schneemann et al. 2020; De Sanctis et al. 2023; Schneemann et al. 2024).

The phenotypic model also makes a naive prediction about how these quantities could change with polyploidization itself, and indeed the rye data suggested that similar $M_{ij}$ and $I_{ij}$ applied for both ploidies, as would be the case if homozygous phenotypic effects were unaffected by polyploidization. Of course, this result is only suggestive, and it seems unlikely that any simple fitness landscape model could capture all the diverse morphological, cytological, and genetic effects of polypoidization, including genome instability (Otto and Whitton 2000; Riddle et al. 2006; Soltis et al. 2014). Still, we hope that our predictions for the masking and dosage effects in polyploids will help to tease these factors apart.

## Figures and Tables

**Fig. B1.**
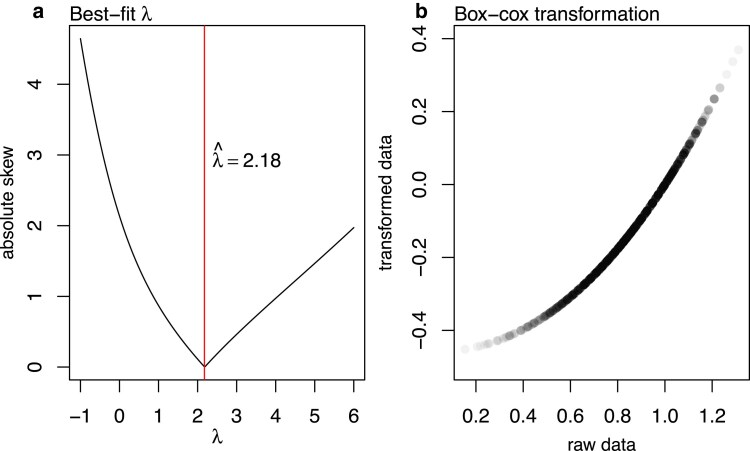
Maize data transformation. a) The best-fit *λ* used in the Box–Cox transformation of the maize data minimizes mean absolute skew per generation. b) The corresponding Box–Cox transformation.

**Fig. B2.**
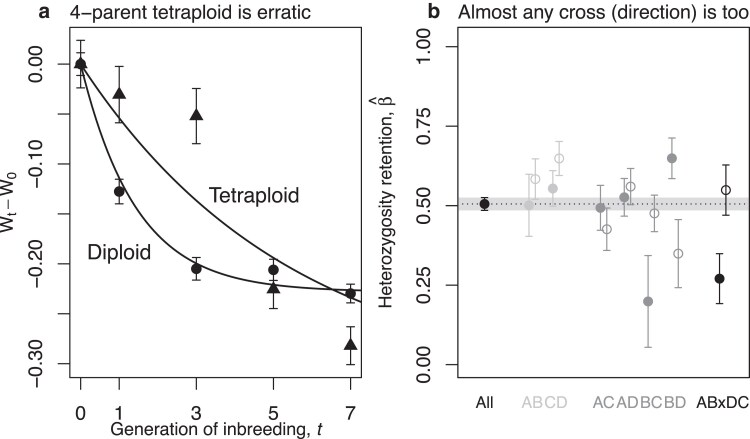
Four-parent tetraploids and cross direction effect in maize. a) The diploid 4-parent cross (∙) fits the model predictions, while the tetraploid (▴) fits poorly and shows an erratic loss of heterosis. Plotting details match Fig. 1a. b) Even for diploid data, estimates of *β* are highly heterogeneous between crosses and cross directions, although the estimate from all diploid data combined very closely fits the prediction of β=1/2 (dotted line). The filled and open points show estimates for the cross listed on the *x*-axis and its reciprocal, respectively.

**Fig. C1.**
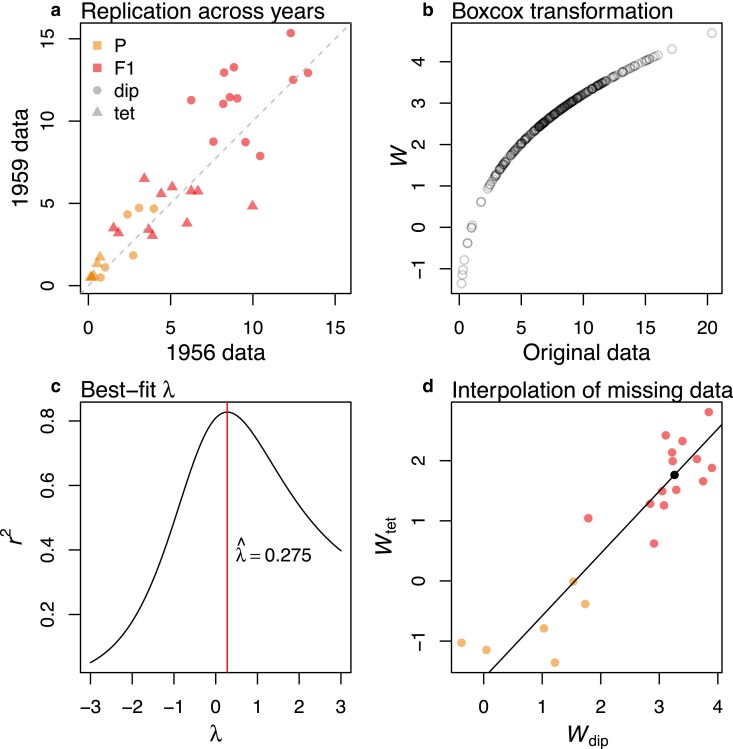
Rye data transformation and interpolation. a) The fitness of diploid and tetraploid (circles and triangles) fixed genotypes (parental lines shown in orange, and F1 in red) is highly correlated between years 1956 and 1959. b) The relationship between the raw and Box–Cox-transformed data. c) The best-fit λ=0.275 (vertical red line) yielded a high correlation in diploid and tetraploid values of fixed genotypes, while other values gave a substantially worse fit. d) With the correlation between the transformed diploid and tetraploid values, we interpolated the missing tetraploid cross (black point) from the best-fit linear model (diagonal line).

**Fig. C2.**
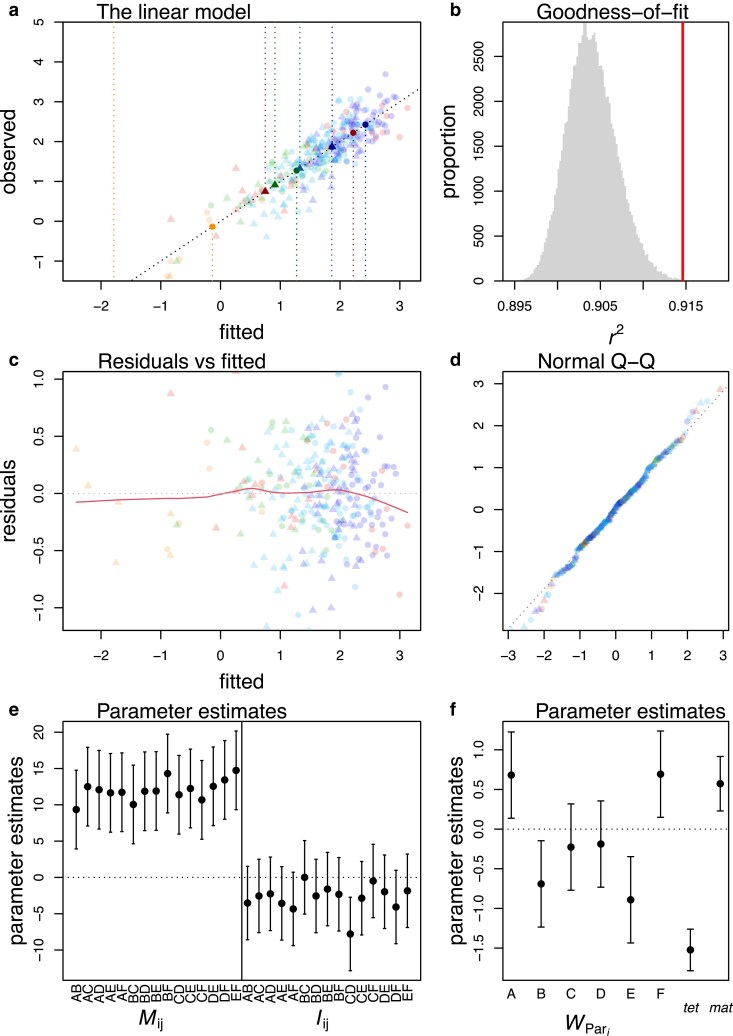
Model fit to rye data. a) The preferred model gives a good fit to the rye data. Faint circles and triangles show the fitted and observed values of each diploid and tetraploid cross. The mean for each cross type is indicated with a clear point and vertical dotted line. b) The $r^{2}$ correlation coefficient of the preferred model (vertical red line) lies outside the distribution obtained from fitting the same model after shuffling the ancestry labels (gray histogram). c) The residuals of the model (*y*-axis) are evenly dispersed across the fitted values (*x*-axis). d) Normal Quantile–quantile plot shows that model residuals are approximately normally distributed (falling on the diagonal). e and f) The parameter estimates under the preferred model indicate that $M_{ij}$ is similar for most parental pairs, while epistasis $I_{ij}$ is on average slightly negative. Parental fitnesses vary, while being tetraploid (tet) brings a significant fitness cost, and having a fit F1 mother (mat) brings a fitness boost.

**Fig. D1.**
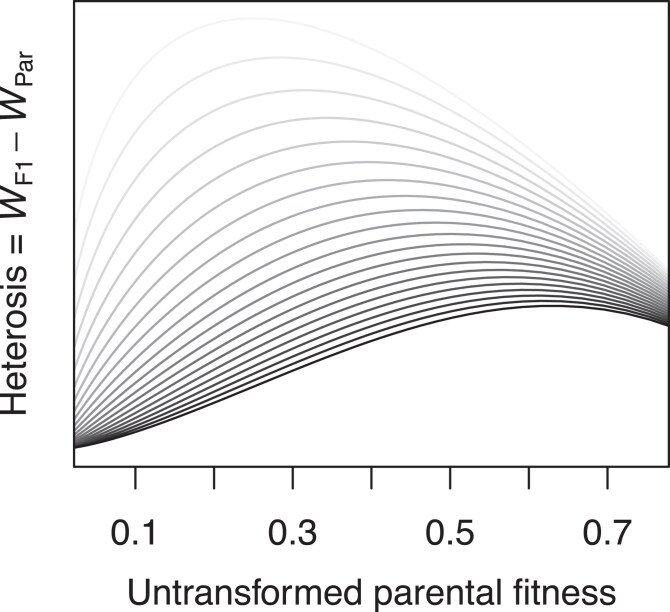
Change in heterosis with parental improvement depends on transformation. For a given parental fitness, heterosis may increase, decrease or remain constant as parental fitness increases, depending on the transformation used. Gray scale indicates *λ* value used in Box–Cox-transformation, from 1 (light gray) to 3 (black).

## Data Availability

The data reanalyzed in this study came from Yao et al. (2020) and Lundqvist (1966). No new data were generated for this study. The scripts used to generate the figures are available at 10.6084/m9.figshare.28660019.v1.

## Appendix A: Derivation of main result

In this Appendix, we derive equation (2), starting with an explicit model of selection on phenotypes. The approach follows Lande (1981), Chevin et al. (2014), and De Sanctis et al. (2023), but is generalized to multiallelic loci, arbitrary ploidy, *K*, and hybrids between an arbitrary number, *P*, of parental lineages of the same ploidy. The model posits that selection acts on several quantitative traits, such that the transformed fitness of any individual is given by

(A1 )
$$\begin{array}{rl} W & =W_{o}-\|z-o{\|}^{2}\equiv W_{o}-\sum_{\tau }^{\mathrm{traits}}{\left(z_{\tau }-o_{\tau }\right)}^{2}, \end{array}$$

where $W_{o}$ is the maximum possible transformed fitness, $z_{\tau }$ is the individual’s value of trait *τ*, and $o_{\tau }$ is this trait’s optimal value. If fitness is log transformed, so that *W* denotes log fitness, then this is a standard model in quantitative genetics (e.g. Lande 1976). Let us note that the fitness function, as written, assumes an equal strength of selection on all traits, and no correlated selection between traits. However, because we make no assumptions about the distribution of allelic effects on each trait, by rotating and scaling the phenotypic axes, all results also apply to a more general case, with variable strength and correlated selection (De Sanctis et al. 2023).

From equation (A1), it follows directly that the expected transformed fitness for any group of individuals (such as a particular class of hybrids) is

(A2 )
$$E\left(W\right)=W_{o}-\sum_{\tau }^{\mathrm{traits}}E^{2}\left(z_{\tau }-o_{\tau }\right)-\sum_{\tau }^{\mathrm{traits}}\mathrm{Var}\left(z_{\tau }\right)$$

To go further, we will now make our three major simplifying assumptions: (1) the genetics of each trait is entirely additive; (2) there are no statistical associations between alleles in the base parental populations; and (3) no linkage disequilibria appear during the formation of hybrids. Assumptions (2) and (3) are required if we are to predict hybrid fitness solely from summary statistics of ancestry, but they neglect linkage disequilibria that might build up by selection or drift. However, (2) holds trivially for parental lines that are highly inbred and genetically homogeneous, such that different alleles are fixed in different lines. Given these assumptions, we can write equation (A2) as

(A3 )
$$E\left(W\right)=W_{o}-\sum_{\tau }^{\mathrm{traits}}E^{2}\left(\sum_{\lambda }^{\mathrm{loci}}z_{\lambda \tau }-o_{\tau }\right)-\sum_{\tau }^{\mathrm{traits}}\sum_{\lambda }^{\mathrm{loci}}\mathrm{Var}\left(z_{\lambda \tau }\right),$$

where $z_{\lambda \tau }$ is the contribution of locus *λ* to trait *τ*.

Because equation (A3) contains simple sums over loci and traits, let us now consider results for a single trait affected by alleles at a single locus (dropping the *λ* and *τ* subscripts). First, let us index the parental populations with *k* (where k=1,2,…,P), and index the alleles at our locus with *i*. Then, let us denote as $a_{i}$ the additive effect on the trait of allele *i*, and denote as $q_{ik}$ the allele frequency of allele *i* in population *k*. Given a ploidy level *K*, the number of alleles carried will be multinomially distributed, with mean $Kq_{ik}$, variance $Kq_{ik}(1-q_{ik})$ and covariance $Kq_{ik}q_{\;jk}$. The mean and variance of the trait in population *k* are therefore

(A4 )
$$\;\begin{array}{rl} \mu_{k} & \equiv E(z{)}_{{\mathrm{Par}}_{k}}=K\sum_{i}^{\mathrm{alleles}}a_{i}q_{ik} \end{array}$$

(A5 )
$$\begin{array}{rl} \nu_{k} & \equiv \mathrm{Var}{\left(z\right)}_{{\mathrm{Par}}_{k}}\\ & =K\sum_{i}^{\mathrm{alleles}}a_{i}^{2}q_{ik}(1-q_{ik})+2\sum_{\;j>i}^{\mathrm{alleles}}a_{\;j}a_{\;j}q_{ik}q_{\;jk}. \end{array}$$

Now, consider a hybrid with ancestry from all *P* parental populations. We will initially assume that we know the proportion of alleles it carries from each parental population, denoted $h_{k}$, but not the specific alleles inherited. The $Kh_{k}$ alleles from population *k* will be sampled independently of those sampled from the other populations. As such, the mean and variance of trait values among hybrids, conditional on the $h_{k}$ are

(A6 )
$$E(z\;|\;h_{1},h_{2},\ldots ,h_{P})=\sum_{k=1}^{P}h_{k}\mu_{k}$$

(A7 )
$$\mathrm{Var}(z\;|\;h_{1},h_{2},\ldots ,h_{P})=\sum_{k=1}^{P}h_{k}\nu_{k}.$$

Next, let us assume that the $h_{k}$ are also unknown, but that—from the crossing scheme—we do know the expected ancestry proportions $\left\langle h_{k}\right\rangle$ and their covariances $\left\langle h_{k}h_{l}\rangle -\langle h_{k}\rangle \langle h_{l}\right\rangle$. With this assumption, we have:

(A8 )
$$\begin{array}{rl} E(z) & =E(E(z\;|\;h_{1},h_{2},\ldots ,h_{P}))\\ & =\sum_{k=1}^{P}\langle h_{k}\rangle \mu_{k} \end{array}$$

and from the law of total variance:

(A9 )
$$\begin{array}{rl} \mathrm{Var}(z) & =E(\mathrm{Var}(z\;|\;h_{1},h_{2},\ldots ,h_{P}))+\mathrm{Var}(E(z\;|\;h_{1},h_{2},\ldots ,h_{P})))\\ & =\sum_{k=1}^{P}\langle h_{k}\rangle \nu_{k}+\mathrm{Var}\left(\sum_{k=1}^{P}h_{k}\mu_{k}\right)\\ & =\sum_{k=1}^{P}\langle h_{k}\rangle \nu_{k}+\sum_{k,l}^{P}\mu_{k}\mu_{l}\left[\langle h_{k}h_{l}\rangle -\langle h_{k}\rangle \langle h_{l}\rangle \right]. \end{array}$$

If we note that all ancestry must come from one of the parental populations, such that $\sum_{k}\langle h_{k}\rangle =1$, then the second term of equation (A9) can also be rewritten as follows

(A10 )
$$\begin{array}{rl} \mathrm{Var}(z)-\sum_{k=1}^{P}\langle h_{k}\rangle \nu_{k} & =\sum_{k,l}^{P}\mu_{k}\mu_{l}\left[\langle h_{k}h_{l}\rangle -\langle h_{k}\rangle \langle h_{l}\rangle \right]\\ & =\sum_{k}^{P}\mu_{k}^{2}\left[\langle h_{k}^{2}\rangle -\langle h_{k}\rangle^{2}\right]\\ & \;+2\sum_{k,l>k}^{P}\mu_{k}\mu_{l}\left[\langle h_{k}h_{l}\rangle -\langle h_{k}\rangle \langle h_{l}\rangle \right]\\ & =\sum_{k=1}^{P}\mu_{k}^{2}\left[\langle h_{k}\rangle (1-\langle h_{k}\rangle )-\langle h_{k}(1-h_{k})\rangle \right]\\ & \;+2\sum_{k,l>k}^{P}\mu_{k}\mu_{l}\left[\langle h_{k}h_{l}\rangle -\langle h_{k}\rangle \langle h_{l}\rangle \right]\\ & =\sum_{k,l\neq k}^{P}\mu_{k}^{2}\left[\langle h_{k}\rangle \langle h_{l}\rangle -\langle h_{k}h_{l}\rangle \right]\\ & \;+2\sum_{k,l>k}^{P}\mu_{k}\mu_{l}\left[\langle h_{k}h_{l}\rangle -\langle h_{k}\rangle \langle h_{l}\rangle \right]\\ & =-\sum_{k,l>k}^{P}(\mu_{k}-\mu_{l}{)}^{2}\left[\langle h_{k}h_{l}\rangle -\langle h_{k}\rangle \langle h_{l}\rangle \right]. \end{array}$$

We can also calculate the second term of equation (A2) as

(A11 )
$$\begin{array}{rl} E^{2}\left(z-o\right) & ={\left(\sum_{k=1}^{P}\langle h_{k}\rangle \mu_{k}-o\right)}^{2}={\left(\sum_{k=1}^{P}\langle h_{k}\rangle (\mu_{k}-o)\right)}^{2}\\ & =\sum_{k,l}^{P}\langle h_{k}\rangle \langle h_{l}\rangle (\mu_{k}-o)(\mu_{l}-o)\\ & =\sum_{k,l}^{P}\langle h_{k}\rangle \langle h_{l}\rangle \frac{(\mu_{k}-o{)}^{2}+(\mu_{l}-o{)}^{2}-(\mu_{k}-\mu_{l}{)}^{2}}{2}\\ & =\sum_{k=1}^{P}\langle h_{k}\rangle^{2}(\mu_{k}-o{)}^{2}\\ & \;+\sum_{k,l>i}^{P}\langle h_{k}\rangle \langle h_{l}\rangle \left((\mu_{k}-o{)}^{2}+(\mu_{l}-o{)}^{2}-(\mu_{k}-\mu_{l}{)}^{2}\right)\\ & =\sum_{k=1}^{P}\langle h_{k}\rangle (\mu_{k}-o{)}^{2}-\sum_{k,l>k}^{P}\langle h_{k}\rangle \langle h_{l}\rangle (\mu_{k}-\mu_{l}{)}^{2}. \end{array}$$

We can now substitute the single-trait single-locus results of equations (A9)–(A11) into equation (A3). To do this, let us define $\mu_{k\lambda \tau }$ ($\nu_{k\lambda \tau }$) as the contribution of locus *λ* to the mean (variance) of trait *τ* in population *k*. Then, the three terms of equation (A3) become

(A12 )
$$\begin{array}{rl} E\left(W\right) & =W_{o}\\ & \;-\sum_{k=1}^{P}[\langle h_{k}\rangle {\left(\sum_{\tau }^{\mathrm{traits}}\sum_{\lambda }^{\mathrm{loci}}\mu_{k\lambda \tau }-o\right)}^{2}\\ & \;-\sum_{l>k}^{P}\langle h_{k}\rangle \langle h_{l}\rangle {\left(\sum_{\tau }^{\mathrm{traits}}\sum_{\lambda }^{\mathrm{loci}}\mu_{k\lambda \tau }-\mu_{l\lambda \tau }\right)}^{2}]\\ & \;-\sum_{k=1}^{P}[\langle h_{k}\rangle \sum_{\tau }^{\mathrm{traits}}\sum_{\lambda }^{\mathrm{loci}}\nu_{k\lambda \tau }\\ & \;-\sum_{l>k}^{P}\left(\langle h_{k}h_{l}\rangle -\langle h_{k}\rangle \langle h_{l}\rangle \right)\sum_{\tau }^{\mathrm{traits}}\sum_{\lambda }^{\mathrm{loci}}(\mu_{k\lambda \tau }-\mu_{l\lambda \tau }{)}^{2}]. \end{array}$$

If we note that $\sum_{k=1}^{P}\langle h_{k}\rangle =1$, then equation (2) of the main text follows by rearranging terms, and defining the following quantities.

(A13 )
$$W_{{\mathrm{Par}}_{k}}=W_{o}-\sum_{\tau }^{\mathrm{traits}}\left[{\left(\sum_{\lambda }^{\mathrm{loci}}\mu_{k\lambda \tau }-o_{\tau }\right)}^{2}+\sum_{\lambda }^{\mathrm{loci}}\nu_{k\lambda \tau }\right]$$

(A14 )
$$M_{kl}\equiv \sum_{\tau }^{\mathrm{traits}}\sum_{\lambda }^{\mathrm{loci}}{\left(\mu_{k\lambda \tau }-\mu_{l\lambda \tau }\right)}^{2}$$

(A15 )
$$m_{kl}=\sum_{\tau }^{\mathrm{traits}}{\left(\sum_{\lambda }^{\mathrm{loci}}\mu_{k\lambda \tau }-\mu_{l\lambda \tau }\right)}^{2}$$

(A16 )
$$I_{kl}\equiv m_{kl}-M_{kl}.$$

Here, equation (A13) is simply the mean transformed fitness of parental population *k*, while equations (A14)–(A15) both quantify the evolutionary change between populations *k* and *l*, via the squared changes in allele frequencies, weighted by allelic effect sizes (see equation (A4)). (Note that, to better allow for arbitrary ploidy, these quantities differ by a factor $K^{2}$ from the earlier single-allele-based definitions in De Sanctis et al. 2023.) These two different measures capture the *total amount* of change ($M_{kl}$) and the *net effect* of the change ($m_{kl}$), with the difference being that a pair of compensatory changes, whose effects cancel each other out, both contribute to the total amount of change, but contribute nothing to the net effect of change (De Sanctis et al. 2023). The difference between these quantities (equation (A16)) therefore measures fitness interactions, by quantifying the amount of compensatory evolution (Chevin et al. 2014; Simon et al. 2018; De Sanctis et al. 2023; Schneemann et al. 2024). Compensatory evolution and coadaptation within the parental lines reduce $m_{kl}$ relative to $M_{kl}$, implying negative fitness interactions when alleles are placed in the “wrong” background such that $I_{kl}<0$. $I_{kl}=0$ will hold if all changes affect different traits, and $E(I_{kl})=0$ will hold if changes are randomly orientated in phenotypic space (De Sanctis et al. 2023). Equations (A14)–(A16) also explain the predictions that $I_{kl}\geq -M_{kl}$ and that $M_{kl}>0$.

To intuit why the masking effect of heterozygosity is weighted by $M_{kl}$, it may help to think of $M_{kl}$ as the summed segregation load of both parents. Consider a scenario where parental lines each accumulated randomly orientated mutations and $I_{kl}$ takes its expected value of 0. In this case, the expected fitness of a randomly chosen parental line is equal to that of a fully homozygous hybrid with a randomly chosen mix of derived alleles. In both cases, we have $E(W)=W_{o}-M_{kl}/2$. The fitness of fully heterozygous hybrids meanwhile is $E(W)=W_{o}-M_{kl}/2+M_{kl}/4=W_{o}-M_{kl}/4$. This shows that the heterozygosity term compensates for the weighted parental fitness term, as heterozygotes are less exposed to the segregation load because deleterious mutations tend to be recessive (Manna et al. 2011).

## Appendix B: Details of reanalysis of Yao et al. (2020) maize dataAppendix B: Details of reanalysis

### Data description

The dataset from Yao et al. (2020) comprises hybrids of four inbred lines of maize (*Zea mays* lines A188, Oh43, W22, and B73), here labeled A, B, C, and D. For each cross, seven generations of selfed offspring are available, with three kernels taken from different ears after one round of selfing, such that subsequent generations include three selfing lineages. All generations of plants were grown simultaneously in a randomized complete block design (across two fields in 2008 and three fields in 2009) and yield-related traits were recorded after 1, 3, 5, and 7 generations of selfing. Yao et al. (2020) reported data for nine distinct yield-related traits (ear length, 4-week, 6-week, and adult height; leaf length and width; tassel branch number; and silk and flower times). Before carrying out any analyses, we chose ear length as the trait closest to overall plant productivity or fitness. On average 45 (min. 3, max. 76), individuals were recorded per year, field, ploidy, cross, and generation combination. The data reported are individual measurements normalized by the value of the corresponding F1 (same cross, ploidy, and year), and measurements of the inbred lines themselves were not included. For both reasons, we could not use these data to estimate $M_{ij}$ or investigate the effect of polyploidization *per se*.

### Model fitting

The maize data showed a large degree of skew in the residuals around their means, violating the assumptions of least-squares model fitting. As such, we used a Box–Cox transformation, replacing the raw measurement *x* with $(x^{\lambda }-1)/\lambda$, and choosing the value of *λ* that minimized the absolute skew (third central moment) averaged over each generation of the data. Figure B1 shows results for the pooled crosses used in the main text (A×B and C×D); these results show the absolute skew of the transformed data with $\hat{\lambda }$ is negligible, but increases rapidly for higher or lower *λ* values. After transformation, we fit our nonlinear model to these data (equation (8)), using the least-squares method implemented in the *R* function *nlsLM* (R Core Team 2021; Elzhov et al. 2022) and calculated confidence intervals with the asymptotic method implemented in the function *confint2* (Fox et al. 2001). To calculate the dosage weighting ratio $\beta_{1\;:\;3}/\beta_{2\;:\;2}$ in Fig. 1e, we used the *R* function *deltaFunction* (Fox et al. 2001) to obtain the maximum likelihood estimate and confidence intervals on the ratio.

### Predictions for four-parent data

Yao et al. (2020) also provided data for other crosses: a four-parent double cross (AB×CD) in both diploid and tetraploid state, and all six of the possible two-parent crosses in diploid state. The diploid crosses were available in both cross directions, while all of the tetraploid crosses (including those in the main text) were present in just one cross direction.

The prediction in equation (8) generalizes readily to crosses with more than two parents if we weight the $M_{ij}$ according to their contribution to the F2 (i.e. weighted by $\langle h_{i}h_{j}\rangle_{0}$, see Table C1). For example, in the diploid double cross four different types of heterozygote are expected at equal frequency (AC, AD, BC, and BD), while in the tetraploid, all six types of heterozygotes are present. As such, we have

(B1 )
$$\begin{array}{rl} & E\left(W_{t}-W_{0}\right)\\ & \;=\left({\left(\frac{1}{2}\right)}^{t}-1\right)\frac{1}{4}\frac{M_{\mathrm{AC}}+M_{\mathrm{AD}}+M_{\mathrm{BC}}+M_{\mathrm{BD}}}{4},\;K=2,\;P=4\\ & \;=\left({\left(\frac{5}{6}\right)}^{t}-1\right)\frac{1}{4}\left(\frac{M_{\mathrm{AC}}+M_{\mathrm{AD}}+M_{\mathrm{BC}}+M_{\mathrm{BD}}}{4}+\frac{M_{\mathrm{AB}}+M_{\mathrm{CD}}}{6}\right),\\ & \;K=4,P=4. \end{array}$$

Thus, predictions for both ploidies can be written in the form $E(W_{t}-W_{0})=(\beta^{t}-1)\frac{1}{4}\bar{M}$ which we can fit to the data.

**Table C1.** Predictor variables for the F2 generation.

Cross	*P*	*K*	$4\langle h_{A}h_{B}\rangle$	$4\langle h_{B}h_{C}\rangle$	$4\sum_{i,j>i}^{P}\langle h_{i}h_{j}\rangle$	$\left\langle h_{A}\rangle \langle h_{B}\right\rangle$	$\left\langle h_{B}\rangle \langle h_{C}\right\rangle$	$\sum_{i,j>i}^{P}\langle h_{i}\rangle \langle h_{j}\rangle$
AB × AB	2	2	$\frac{1}{2}$	–	$\frac{1}{2}$	$\frac{1}{4}$	–	$\frac{1}{4}$
		4	$\frac{5}{6}$	–	$\frac{5}{6}$	$\frac{1}{4}$	–	$\frac{1}{4}$
AB × AC	3	2	$\frac{1}{4}$	$\frac{1}{4}$	$\frac{3}{4}$	$\frac{1}{8}$	$\frac{1}{16}$	$\frac{5}{16}$
		4	$\frac{5}{12}$	$\frac{1}{4}$	$\frac{13}{12}$	$\frac{1}{8}$	$\frac{1}{16}$	$\frac{5}{16}$
AB × CD	4	2	0	$\frac{1}{4}$	1	$\frac{1}{16}$	$\frac{1}{16}$	$\frac{3}{8}$
		4	$\frac{1}{6}$	$\frac{1}{4}$	$\frac{4}{3}$	$\frac{1}{16}$	$\frac{1}{16}$	$\frac{3}{8}$

Note the linear increase in the total heterozygosity with *P*, depicted in Fig. 2a.

Figure B2a shows this prediction fit to the four-parent data (diploid: circles; and tetraploid: triangles), Box–Cox transformed with $\hat{\lambda }=2.18$ as above. Results show that the diploid points, which are averaged over the two cross directions (AB×DC and DC×AB) fit the prediction fairly well. By contrast, the tetraploid 4-parent cross, which was available in just one cross direction (BA× CD) fits very poorly. In fact, this cross shows an erratic patterns of loss of heterosis, with little change between generations 1 and 3, but a steep drop in fitness between 3 and 5.

This result might be a true failure of the model, or simply noise in the data. A general impression from the maize analyses is that, even with this large dataset, patterns in the data are relatively noisy unless we average across crosses and/or cross directions (see Fig. 1b and d), which were not available for 4-parent tetraploids. For comparison, Fig. B2b shows *β* estimates for each diploid cross (indicated along the *x*-axis) and direction (filled vs open points). Only when we average over these crosses do we find that our estimate of *β* matches the prediction of 1/2 very closely, while individual estimates can deviate substantially from this value. This prediction of β=1/2 stems purely from Mendelian segregation, rather than any assumptions of the fitness landscape, suggesting that the deviation of the tetraploid 4-parent from model predictions might similarly be a result of sampling noise.

## Appendix C: Details of reanalysis of Lundqvist (1966) rye dataAppendix C: Details of reanalysis

### Data description

The dataset from Lundqvist (1966) involves six inbred parental lines of diploid rye (*Secale cereale*; steel variety produced through 25 generations of selfing). These were treated with colcichine to obtain genotypically matched tetraploid lines, but the plants that underwent treatment were not themselves the parents used in the crosses such that we can ignore the immediate effect of colchicine on fitness. Only the mean and sample size of each cross are reported, and no reciprocal crosses were made. As much as possible, Lundqvist (1966) did attempt to use the same cross direction for the diploids and tetraploids of a given cross, but this was not always possible. The inbred parental lines and F1 were replicated during two experiments in 1956 and 1959 (another attempt was made in 1957 but this failed), whereas the F2 generation was recorded only in 1959. As the 1956 and 1959 replicates generally show a good correlation (see Fig. C1a), we take sample-size weighted averages across these 2 years.

Lundqvist (1966) recorded nine traits for each plant, namely number of spikelets of best-developed ear, seed-setting percentage of best-developed ear, mean kernel weight of best-developed ear, diameter of thickest region of biggest straw, plant height, straw weight, mean and total kernel yield, and number of spikes. Before analyzing the data, we chose “total kernel yield” as the best available proxy for the plant’s overall productivity or fitness.

### Data transformation and interpolation

We transform all the data to find a scaling that generates the strongest correlation between the diploid and tetraploid values, but using only the fixed genotypes (i.e. the inbred parental lines and F1s). In particular, we test a range of values of the scaling parameter *λ* in a Box–Cox transformation, using $W=\frac{(\mathrm{Total}\;\mathrm{Kernel}\;\mathrm{Yield}{)}^{\lambda }-1}{\lambda }$. Then, we regress these Box–Cox-transformed diploid and tetraploid values against each other using SMA regression using $r^{2}={\mathrm{Cor}}^{2}(x+y,x-y)$ to measure goodness of fit (Warton et al. 2012). As we only have the line means and not individual-level measurements for these rye data, this transformation ignores the discrepancy between $E[\frac{x^{\lambda }-1}{\lambda }]$ and $\frac{E{\left(x\right)}^{\lambda }-1}{\lambda }$. Results reported in Fig. C1b,c show that the best-fit *λ* yields a strong correlation between diploid and tetraploid values ($r^{2}=0.83$), while other *λ* values give a weaker correlation. After the transformation, we use the best-fit SMA regression line to interpolate the fitness value of the missing data point: a tetraploid F1 (black point in Fig. C1d). As reported in Fig. 2, we found that the best-fit SMA regression of the transformed diploid and tetraploid values has a slope close to 1 (1.03) and a negative intercept (−1.62). This suggests that the effect of tetraploidy under this scaling is to induce a constant fitness cost.

### Model fitting

To assess the suitability of our model to explain the patterns in this dataset, we fit our model to the full dataset of 282 line means (6 parental lines, 15 F1s and 120 F2s, each in diploid and tetraploid state), using the *lm* function from the *stats* package in R (R Core Team 2021). As our predictor variables, we use the expected ancestry proportions $\left\langle h_{i}\right\rangle$, their pairwise products $\left\langle h_{i}\rangle \langle h_{j}\right\rangle$, and pairwise heterozygosities $\left\langle h_{i}h_{j}\right\rangle$ following from equation (14). For the F2 generation, these are listed in Table C1.

We fit three models to the data, listed in Table C2. In model 1 we estimated ploidy-specific $W_{{\mathrm{Par}}_{i}}^{\left(K\right)}$, $M_{ij}^{\left(K\right)}$, and $I_{ij}^{\left(K\right)}$ parameters, while in model 2 and 3 we estimated shared parameters $W_{{\mathrm{Par}}_{i}}$, $M_{ij}$, and $I_{ij}$ and a separate tetraploidy effect. In model 3, we also included a maternal effect specific to the F2 generation. We compare their fit using the corrected Akaike Information Criterion (AICc), which is suitable for parameter-rich models (Burnham and Anderson 2002). This comparison indicates that the ploidy-specific parameters in model 1 do not provide a significantly improved fit to the data (AICc of 394.33 vs 385.33, where lower is better; see top two rows of Table C2). Adding a maternal effect that affects the F2 generation does significantly improve model fit (385.33 vs 376.06; compare model 2 to model 3 on bottom rows of Table C2). This is consistent with the observation in Fig. 2d that the F2 are shifted upwards along the diagonal line.

**Table C2.** Linear model specifications.

Model	Formula and description	− log (*L*)	AICc	df
**Model 1:** Ploidy-specific effects	lm(W ∼ 0 + HybridIndex:Ploidy + Admixture:Ploidy + Heterozygosity:Ploidy)	98.19	394.33	72
	Estimates a separate set of coefficients $W_{{\mathrm{Par}}_{i}}^{\left(K\right)}$, $M_{ij}^{\left(K\right)}$ and $I_{ij}^{\left(K\right)}$ for diploids and tetraploids.			
**Model 2:** Shared effects across ploidy	lm(W ∼ 0 + HybridIndex + Admixture + Heterozygosity + Ploidy)	148.68	385.55	37
	Estimates a shared set of coefficients $W_{{\mathrm{Par}}_{i}}$, $M_{ij}$ and $I_{ij}$ for diploids and tetraploids, and a tetraploidy effect.			
**Model 3:** Shared effects across ploidy + maternal effect	lm(W ∼ 0 + HybridIndex + Admixture + Heterozygosity + Ploidy + Maternal)	142.58	376.06	38
	Estimates a shared set of coefficients $W_{{\mathrm{Par}}_{i}}$, $M_{ij}$, and $I_{ij}$ for diploids and tetraploids, a tetraploidy effect, and a maternal effect.			

**Predictor variables:**

– HybridIndex: 282×6 matrix of ancestry proportions $\left\langle h_{i}\right\rangle$

– Admixture: 282×15 matrix of pairwise ancestry proportions $\left\langle h_{i}\rangle \langle h_{j}\right\rangle$

– Heterozygosity: 282×15 matrix of pairwise heterozygosities $\left\langle h_{i}h_{j}\right\rangle$

– Ploidy: factor indicating whether cross is tetraploid

– Maternal: factor indicating whether cross is F2

The parameter estimates from our preferred model 3 are depicted in Fig. C2. This figure confirms the adequacy of this model for the rye data, giving a strong correlation of fitted to observed values (Fig. C2A), high goodness-of-fit relative to permutations of the ancestry labels (Fig. C2B), and constant and approximately normally distributed residuals (Fig. C2C and D). Moreover, this model produces sensible parameter estimates. The estimates of $M_{ij}$ are all large and significantly positive with slight variation among pairs of lines, while $I_{ij}$ estimates are slightly negative but mostly not significantly different from zero (Fig. C2e). This is exactly predicted when parental lines diverged under random drift with possibly very weak stabilizing selection (De Sanctis et al. 2023; Schneemann et al. 2024). As anticipated, we also find a significant fitness cost associated with tetraploidy (denoted *tet* in Fig. C2f and Ploidy in Table C2), and a beneficial maternal effect acting on the F2 generation (denoted *mat* in Fig. C2f and Maternal in Table C2). Figure C3 further shows the diploid vs tetraploid F2 breakdown, where the fit to predictions is substantially improved after accounting for the maternal effect estimate (compare red and black line).

## Appendix D: Heterosis and parental improvement

Birchler (2013) suggested that the dominance theory of heterosis implies that the level of heterosis should decrease as parental lines increase in fitness (e.g. through purging their deleterious mutations). This pattern was indeed observed by Duvick (2005) in maize for F1 hybrids between lines stemming from different decades of vast yield improvement (although see Troyer and Wellin 2009). However, the relationship between the level of heterosis and parental fitness depends on the transformation of the data. Figure D1 illustrates increasing parental fitness along the *x*-axis, and the predicted level of heterosis under our model on the *y*-axis, for different Box–Cox-transformations i.e. values of *λ* (compare light to dark curves). In all cases, the level of heterosis first increases faster than parental fitness, reaches a maximum, and then decreases with parental fitness. The latter phase is described verbally by Birchler (2013). Yet, the range of parental fitness where each of these regimes occurs depends on the data transformation. Hence, data of this kind do not provide a simple proof for or against the dominance theory as an explanation for heterosis.
